# Donor-derived cell-free DNA predicted allograft rejection and severe microvascular inflammation in kidney transplant recipients

**DOI:** 10.3389/fimmu.2024.1433918

**Published:** 2024-07-09

**Authors:** Hyung Duk Kim, Hyunjoo Bae, Hyunhye Kang, Hanbi Lee, Sang Hun Eum, Chul Woo Yang, Yeong Jin Choi, Byung Ha Chung, Eun-Jee Oh

**Affiliations:** ^1^ Division of Nephrology, Department of Internal Medicine, Eunpyeoung St. Mary’s Hospital, College of Medicine, The Catholic University of Korea, Seoul, Republic of Korea; ^2^ Department of Biomedicine & Health Sciences, The Catholic University of Korea, Seoul, Republic of Korea; ^3^ Department of Laboratory Medicine, Seoul St. Mary’s Hospital, College of Medicine, The Catholic University of Korea, Seoul, Republic of Korea; ^4^ Research and Development Institute for In Vitro Diagnostic Medical Devices of Catholic University of Korea, Seoul, Republic of Korea; ^5^ Division of Nephrology, Department of Internal Medicine, Seoul St. Mary’s Hospital, College of Medicine, The Catholic University of Korea, Seoul, Republic of Korea; ^6^ Division of Nephrology, Department of Internal Medicine, Incheon St. Mary’s Hospital, College of Medicine, The Catholic University of Korea, Seoul, Republic of Korea; ^7^ Department of Hospital Pathology, Seoul St. Mary’s Hospital, College of Medicine, The Catholic University of Korea, Seoul, Republic of Korea

**Keywords:** kidney transplantation, cell-free nucleic acids, allograft rejection, microvascular rejection, tissue donors

## Abstract

**Introduction:**

The aim of this study is to investigate the clinical validity of donor-derived cell-free DNA (dd-cfDNA) in comparison with that of donor specific anti-HLA antibody (DSA) for predicting biopsy-proven rejection (BPR)and severe microvascular inflammation (severe MVI) in kidney transplant recipients (KTRs).

**Methods:**

In this prospective observational investigation, 64 KTRs who underwent the indicated biopsies were included. Blood samples collected prior to biopsy were tested for dd-cfDNA and DSA. Biopsy specimens were classified by a renal pathologist according to the Banff classification. The predictive performance of dd-cfDNA and DSA for histological allograft diagnosis was assessed.

**Results:**

KTRs were categorized into the high and low dd-cfDNA groups based on a level of 0.4%. Eighteen patients (28.1%) had positive DSA at biopsy, exhibiting higher dd-cfDNA levels than the DSA-negative patients. BPR and severe MVI incidences were elevated in the high dd-cfDNA group (BPR: 42.9% vs. 3.4%, P <0.001; severe MVI: 37.1% vs. 3.4%, P = 0.001). Also, elevated glomerulitis and MVI scores were observed in the high dd-cfDNA group. DSA showed the highest predictive value for BPR (AUC = 0.880), whereas dd-cfDNA alone excelled in predicting severe MVI (AUC = 0.855). Combination of DSA and dd-cfDNA (>0.4%) yielded sensitivities of 80.0% and 50.0% with specificities of 90.7% and 88.0% for antibody-mediated rejection and severe MVI detection, respectively.

**Conclusion:**

The dd-cfDNA test is a predictive tool for BPR and severe MVI, and it can improve the performance, especially when combined with DSA for BPR.

## 1 Introduction

Kidney transplantation (KT) is the most ideal modality for the treatment of end stage renal disease (ESRD) patients (1). However, allograft rejection is still an important obstacle for successful long-term maintenance of allograft function. Therefore, early detection and management of acute rejection is important in KT recipients (KTRs). Current diagnostic approaches primarily rely on kidney allograft biopsies, which is the gold standard to evaluate allograft injury. However, kidney allograft biopsy is an invasive procedure associated with the risk of bleeding, and it has several limitations, such as sampling variability, high cost, difficulties of serial use, and delayed results. Thus, there is an increasing need for minimally invasive biomarkers that can facilitate early diagnosis of allograft rejection in KTRs.

Application of Luminex single antigen (LSA) assay for the detection of donor-specific anti-HLA antibody has led to remarkable advances in minimally invasive biomarker development (2). Accumulating evidence has revealed that donor-specific anti-HLA antibody (DSA) plays a critical role in the progression of antibody-mediated allograft tissue injury, which in turn, is the most important cause of late allograft failure in KTRs (3–5). Therefore, detection and monitoring of DSA may help in predicting allograft outcomes and planning proper management to prevent decline of allograft function in KTRs (6, 7). However, it is important to note that not all DSA-positive patients develop antibody-mediated rejection (ABMR). According to recent reports, approximately half of the DSA-positive patients are diagnosed with ABMR in general and cases of DSA-negative ABMR are also found (8, 9). Therefore, the clinical validity of DSA detection has limitations in its role as a method for supporting the decision to perform a kidney allograft biopsy. Meanwhile, donor-derived cell-free DNA (dd-cfDNA) has emerged as a promising non-invasive biomarker for the detection of allograft injury, particularly endothelial injury as seen in ABMR and rejection (10). dd-cfDNA comprises a fraction of circulating cell-free DNA in the recipient’s bloodstream, originating from the donor-derived cells of kidney allograft (11). The release of dd-cfDNA into the circulation during episodes of allograft injury presents a unique opportunity for early detection and timely intervention. Recent prospective studies have shown a significant correlation between dd-cfDNA levels and allograft injury and rejection (12–15). Particularly, elevated dd-cfDNA levels have shown a strong association with the occurrence of ABMR (12–15). Additionally, persistently high dd-cfDNA levels have been found to predict the development of *de novo* DSA and a decline in the estimated glomerular filtration rate (eGFR) of more than 25% (14).

Based on the background presented above, we hypothesized that elevated levels of dd-cfDNA could serve as a predictive indicator for significant histological changes. Consequently, this study aims to investigate the potential clinical and histological correlations of dd-cfDNA at the time of biopsy. The main objectives of this study were to evaluate the prognostic capability of dd-cfDNA in anticipating the occurrence of various types of rejection, including any rejection and severe microvascular inflammation (MVI) and to assess its predictive capacity in gauging the extent of histological damage using the Banff score. The supplementary aim of the study was to perform a comparative analysis of dd-cfDNA with other predictive parameters, such as DSA.

## 2 Methods

### 2.1 Study population

This prospective observational investigation included KTRs who underwent allograft biopsies as clinically indicated during the period from March 1, 2021 to April 22, 2024, at Seoul St. Mary’s Hospital.

We excluded patients who received kidney transplants more than twice, multiple solid organ transplant recipients, such as liver transplant recipients, and bone marrow transplant recipients, pregnant patients, and patients who received a kidney from their identical twin donors, which makes it difficult to distinguish between dd-cfDNA and recipient-derived cfDNA. In addition, only KTRs who had completed a minimum of three months post-transplantation were included in this study, considering the dynamics of cfDNA in the early stages of transplantation without any rejection mechanism.

Blood samples for dd-cfDNA analysis were collected just before the biopsy procedure from all participants. Simultaneous collection of blood and urine samples was performed during the dd-cfDNA assay, with additional tests conducted for serum creatinine, CKD-EPI eGFR, proteinuria, and DSA. All participants provided written informed consent in accordance with the Declaration of Helsinki. This study was approved by the local Institutional Review Board of Seoul St. Mary’s Hospital (KC19TESI0096 and KC19TESI0043).

### 2.2 Allograft biopsy and histological assessment

Clinically indicated biopsies were performed in cases of renal dysfunction, increase in the preformed DSA MFI values, or development of *de novo* DSA. Renal dysfunction was assessed based on clinical indicators, such as elevated serum creatinine, decreased eGFR, or the occurrence or increase of proteinuria, according to the decision of the transplant clinicians. Allograft biopsy was performed under ultrasound guidance by a trained nephrologist using a 16-gauge needle. At least two tissue cores were obtained, and the histopathological diagnosis was made according to the Banff Working Group criteria (16). ABMR and T-cell-mediated rejection (TCMR) were diagnosed according to the Banff criteria. We also scored each individual pathological component of ABMR and TCMR separately to test for the association with dd-cfDNA levels. The glomerulitis score (g score) plus peritubular capillaritis score (ptc score) was referred as the MVI score. Regardless of DSA, a MVI score ≥3 was defined as severe MVI. And severe MVI without DSA was defined as microvascular rejection (MVR) (17). Allograft biopsies were evaluated by a renal pathologist who was blinded to the measurement of dd-cfDNA levels.

### 2.3 dd-cfDNA assay

The assessment of dd-cfDNA levels began with the collection of 10 cc of whole blood using a Streck cfDNA tube. Subsequent cfDNA extraction involved the utilization of the QIAamp DSP Circulating NA Kit (QIAGEN, Hilden, Germany) along with the QIAvac 24 system (QIAGEN, Hilden, Germany). The quality of the cfDNA was evaluated using the Agilent 2100 Bioanalyzer (Agilent), and its concentration was determined using the Qubit™ dsDNA 1x HS Assay Kit (Thermo Fisher). The extracted cfDNA was diluted in nuclease-free water to achieve a final concentration of 10 ng/μL in a total volume of 16 μl. cfDNA libraries were generated using the AlloSeq cfDNA kit (CareDx, Brisbane, CA, USA). In a PCR plate, 4 μL of the forward index primers (i5) were added to the respective sample wells, guided by the plate layout specified in the Sample Sheet. Similarly, 4 μL of the reverse index primers (i7) were added to the appropriate sample wells. Next, 16 μL of the prepared DNA was added to each sample well, followed by creation of a Master Mix in a 1.5 ml tube for the required number of samples. The recommended quantities of the AlloSeq cfDNA PCR Mix, AlloSeq cfDNA PCR Enzyme, and AlloSeq cfDNA SNP Primer Pool were used. Using the Master Mix, PCR was performed on the VeritiTM thermal cycler (Thermo Fisher), following the PCR cycling protocol. The concentration of PCR-amplified cfDNA was quantified using the Qubit™ dsDNA 1x HS Assay Kit (Thermo Fisher), followed by the PCR product clean-up procedure. The library pool produced during this clean-up process could have a concentration as low as 0.3 ng/μl. Sequencing was performed on the MiSeq instrument (Illumina, San Diego, CA, USA) using the MiSeq Reagent Kit v3 (150 cycle) with a 2x75 bp cycle configuration. The final loading concentration for sequencing was set at 20 pM, including a 1% PhiX control. The FASTQ files were analyzed, and the %dd-cfDNA was calculated using the AlloSeq cfDNA Software (IFU085-1 v1.4 08.2020) as the publicly available analytical validation of AlloSeq cfDNA (18) only focused on the %dd-cfDNA values between 0.25% and 12%.

### 2.4 HLA antibody detection with Luminex single antigen assay

LABScreen Single Antigen assays (One Lambda Inc., A Thermo Fisher Scientific Brand, Canoga Park, CA, USA) were performed to detect HLA antibodies. The positive criterion was a mean fluorescence intensity (MFI) level ≥ 1000. In all patients and donors, HLA-A, B, C, DRB1, and DQB1 typing were performed using the SSO-LABType commercial kit (One Lambda, Inc., Canoga Park, CA, USA). DQA1 type test was conducted only when necessary for further interpretation of DSA. If the anti-HLA antibody detected in the patient corresponded to the HLA type of the donor, it was classified as DSA-positive.

### 2.5 Statistical analyses

Statistical analyses were performed with SPSS version 25.0 (SPSS, Chicago, IL, USA). Continuous variables are presented as the mean ± standard deviation (SD) or median (interquartile range, IQR). The *t*-tests were used for analysis of continuous variables with a normal distribution, and the Mann-Whitney test was used for variables with non-normal distribution. The average categorical variables are presented as counts and percentages. Logistic regression analysis and Receiver Operating Characteristic (ROC) curve analysis were used to compare the predictive performance of clinical parameters and dd-cfDNA for biopsy-proven rejection (BPR) and severe MVI. DSA is already known as a risk factor for BPR; thus, the combination of dd-cfDNA and DSA was included in the ROC curve analysis to assess whether the predictive performance could be improved. The chi-square and Fisher’s exact tests were used for categorical variables. A *p* value < 0.05 was considered significant.

## 3 Results

### 3.1 Clinical and laboratory characteristics according to the dd-cfDNA levels

A total of 64 KTRs were enrolled in the study at a median 39.5 months post-transplant (IQR: 6.0 – 122.0). The median value of the dd-cfDNA level was 0.49% (IQR: 0.20– 1.09), while 25.0% of the recipients had a dd-cfDNA level more than 1%. Using 0.4% as a threshold, we categorized the cohort of 64 patients into the following two groups: the low cfDNA group (n =29) and the high cfDNA group (n = 35). Baseline characteristics on comparison between the low- and high cfDNA groups are shown in **Table 1**. There was no significant difference in age, sex, post-transplantation period, serum creatinine, eGFR calculated by CKD-EPI, and the amount of proteinuria in a random urine sample between the two groups. There was no difference between the two groups in the proportion of pretransplant DSA-positive patients, ABO incompatibility, and HLA mismatch numbers between the donors and recipients. Eighteen patients (28.1%) had positive DSA at the time of biopsy, and they tended to have higher dd-cfDNA levels compared to the thirty DSA-negative patients (median (IQR): 0.71(0.39 – 1.60) vs. 0.36 (0.18 – 0.81), P = 0.057). The proportion of patients with positive DSA also tended to be higher in the high cfDNA group compared to the low cfDNA group (37.1% versus 17.2%, respectively, P=0.08) (**Table 1**). Out of the 18 DSA-positive patients, 14 individuals exhibited the presence of *de novo* DSA. Furthermore, in patients with *de novo* DSA, notably higher levels of dd-cfDNA were observed when compared to the levels in those without *de novo* DSA (median (IQR): 1.05 (0.39 – 2.00) vs. 0.39 (0.18 – 0.72), P=0.038).

**Table 1:** Baseline characteristics according to the level of dd-cfDNA at the time of biopsy.

	Low dd-cfDNA group (N=29)	High dd-cfDNA group (N=35)	P value
Age (years)	48.6 ± 11.6	52.5 ± 10.9	0.171
Sex (male)	23 (79.3%)	21 (60.0%)	0.100
KT vintage (months)	75.2 ± 84.0	68.5 ± 72.0	0.973
Donor type (living donor)	25 (86.2%)	29 (82.9%)	0.919
Pre-KT DSA (positive)	3 (10.3%)	4 (11.4%)	0.859
ABO incompatibility	7 (24.1%)	14 (40.0%)	0.182
Mismatch number	3.6 ± 1.4	3.6 ± 1.8	0.988
Serum Cr (mg/dL)	2.3 ± 1.0	2.0 ± 0.9	0.117
CKD-EPI eGFR (ml/min/1.73m^2^)	39.6 ± 19.3	45.7 ± 23.6	0.296
Urine PCR (g/gCr)	1.5 ± 2.5	1.3 ± 1.6	0.349
dd-cfDNA (%)	0.2 ± 0.08	1.6 ± 1.4	<0.0001
DSA at Graft Bx.	5 (17.2%)	13 (37.1%)	0.080

Data are expressed as mean ± standard deviation, median (interquartile range), or number (percentage).

cfDNA, cell free DNA; KT, kidney transplantation; DSA, donor-specific anti-HLA antibody; PCR, protein to creatinine ratio.

### 3.2 Biopsy-proven rejection according to the dd-cfDNA level or DSA detection

Out of the 64 KTRs included in the study, 16 patients were confirmed to have experienced rejection through biopsy. Specifically, 9 patients exhibited ABMR, 6 patients presented with TCMR, and 1 patient displayed mixed rejection characterized by both acute ABMR and chronic active TCMR (**Supplementary Figure S1**). Among a total of 10 patients with ABMR, including 1 patient with mixed rejection, 5 were diagnosed with acute ABMR and the other 5 were diagnosed with chronic active ABMR. Furthermore, among the 7 TCMR patients, including 1 patient with mixed rejection, 2 were diagnosed with chronic active TCMR grade IA, 3 with chronic active TCMR grade IB, and the remaining 2 had acute TCMR grade IA and IB. Severe MVI was observed in 14 patients. Among the 48 patients without rejection, the most common pathologic diagnosis was glomerulonephritis, confirmed in a total of 21 patients (7 with IgA nephropathy, 6 with chronic sclerosing glomerulonephritis, and 3 with focal segmental glomerulosclerosis). Other diagnoses such as diabetic nephropathy, BK virus-associated nephropathy, and acute tubular necrosis were also identified.

**Figure 1:**
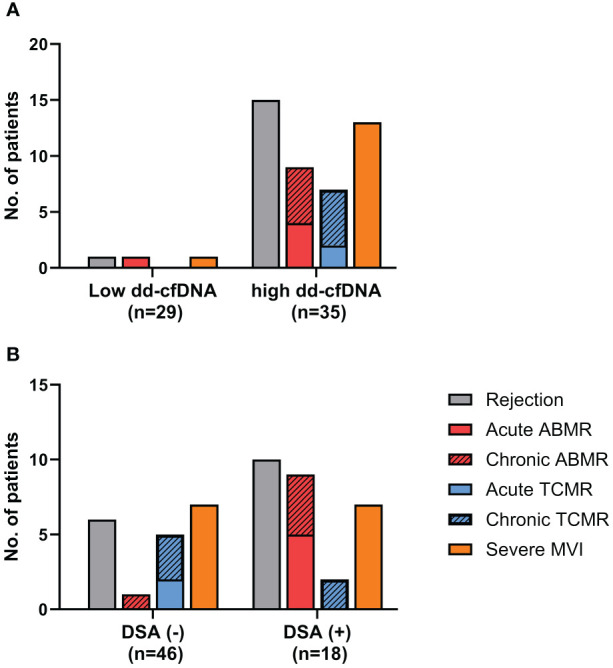
Analysis of the biopsy-proven rejection incidence. **(A)** The high dd-cfDNA group had a significantly higher incidence of biopsy-proven rejection compared to the low dd-cfDNA group (42.9% vs. 3.4%, P<0.001). **(B)** DSA positive (+) patients showed a significantly higher incidence of biopsy-proven rejection than DSA negative **(-)** patients (55.6% vs. 130%, p<0.0001). ABMR, Antibody-mediated rejection; TCMR, T cell mediated rejection; MVI, microvascular inflammation; +, positive; -, negative.

When comparing the frequencies of rejection between the high dd-cfDNA group and the low dd-cfDNA group, the incidence of rejection was significantly higher in the high dd-cfDNA group (15/35, 42.9%) as opposed to the low dd-cfDNA group (1/29, 3.4%) (P<0.001). Specifically, the high dd-cfDNA group had 15 cases of rejection, consisting of 9 cases of ABMR, 7 cases of TCMR, and 1 case of mixed rejection. In contrast, the low dd-cfDNA group had only one case of rejection, associated with ABMR. On comparison of severe MVI, the high dd-cfDNA group had thirteen patients with severe MVI, while low dd-cfDNA group had only one case of severe MVI (13/35, 37.1% versus 1/29, 3.4%, P = 0.001) (**Figure 1A**).

**Figure 2:**
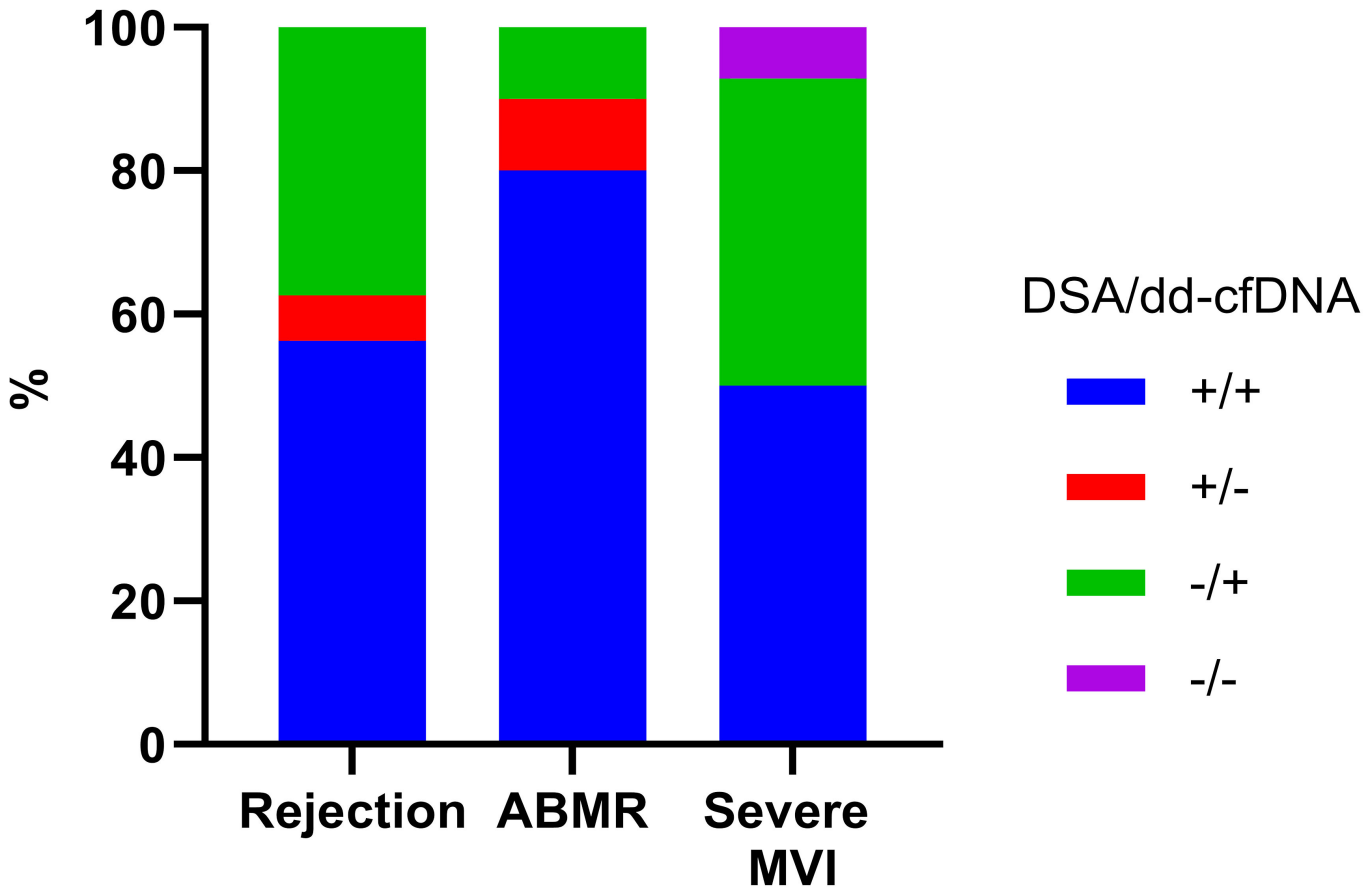
DSA and dd-cfDNA results according to the biopsy-proven rejection type. DSA, donor-specific antibody; ABMR, Antibody-mediated rejection; MVI, microvascular inflammation; +, positive; -, negative.

On comparison of the rejection frequency between 18 cases in the DSA-positive group and 46 cases in the DSA-negative group, the DSA-positive group had a significantly higher frequency of rejection (10/18, 55.6% vs. 6/46, 13.0%, p<0.0001) (**Figure 1B**). In DSA-positive patients, 9 patients had ABMR, 2 patient had TCMR, and the other 1 patient had mixed rejection. In the patients without DSA, there was 1 case of ABMR and 5 cases of TCMR. The proportion of patients diagnosed with severe MVI was also higher among the DSA-positive patients (7/18, 38.9% vs. 7/46,15.2%, p=0.04). When we analyzed the distribution of DSA and dd-cfDNA results in patients with rejection, ABMR and severe MVI, DSA/dd-cfDNA (+/-) cases were found in 6.3% of rejection and 10% of ABMR, while DSA/dd-cfDNA (-/+) cases were found in 37.5% of rejection, 10% of ABMR, and 42.9% of severe MVI (**Figure 2**).

### 3.3 Banff score according to the dd-cfDNA level or DSA detection

**Figure 3:**
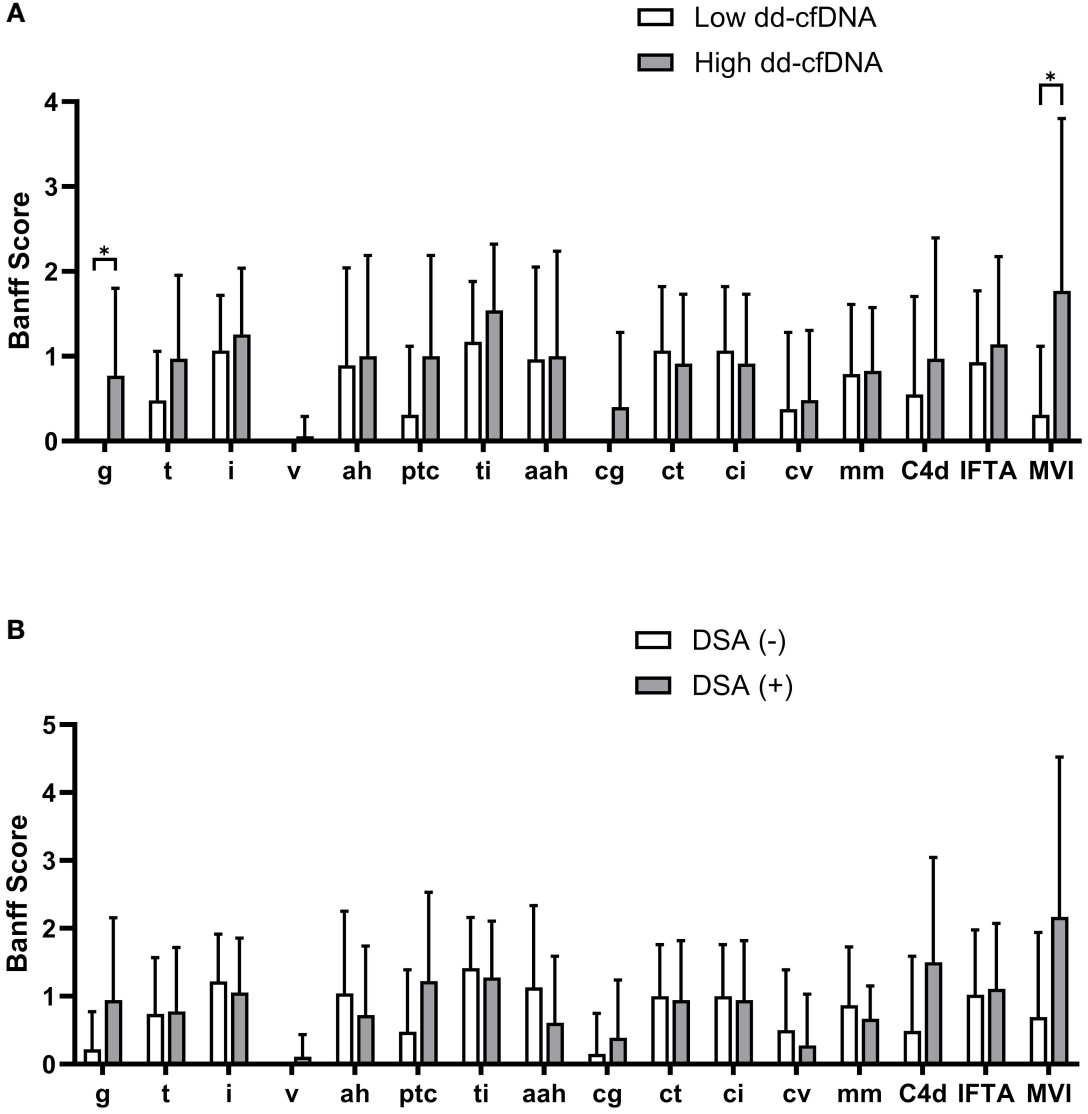
Comparison of Banff scores according to the dd-cfDNA and DSA results. Each bar represents average Banff scores along with their standard deviation. The high dd-cfDNA group showed higher glomerulitis and peritubular capillaritis scores compared to the low dd-cfDNA group **(A)**. The DSA positive patients showed higher scores for glomerulitis, peritubular capillaritis, and C4d on allograft biopsy compared to the DSA negative patients **(B)**. Banff score lesions: g, glomerulitis; t, tubulitis; i, interstitial inflammation; v, arterial inflammation; ah, arteriolar hyalinosis; ptc, peritubular capillaritis; ti, total inflammation; cg, transplant glomerulopathy; ct, tubular atrophy; ci, interstitial fibrosis; cv, arterial intimal thickening; mm, mesangial matrix increase; C4d, C4d deposition; IFTA, interstitial fibrosis and tubular atrophy; MVI, microvascular inflammation. *P<0.05.

We then analyzed the dd-cfDNA levels and DSA according to the Banff elementary lesions. On comparison of Banff scores between the high and low dd-cfDNA groups, the glomerulitis (g) score and peritubular capillaritis (ptc) score were significantly higher in the high dd-cfDNA group (**Figure 3A**). Although the tubulitis (t), total inflammation (ti), transplant glomerulopathy (cg), and C4d deposition (C4d) scores showed a trend towards higher values in the high cfDNA group, the differences did not reach statistical significance. However, the g and MVI score was significantly higher in the high dd-cfDNA group (P<0.001 and p=0.006, respectively). When examining Banff scores based on DSA detection at the time of biopsy, there was a trend towards higher g-score, ptc score, C4d score, and MVI score in DSA-positive patients, but no statistically significant differences were found (p>0.05) (**Figure 3B**).

### 3.4 Comparison of dd-cfDNA levels according to the histologic findings at the time of biopsy

**Figure 4:**
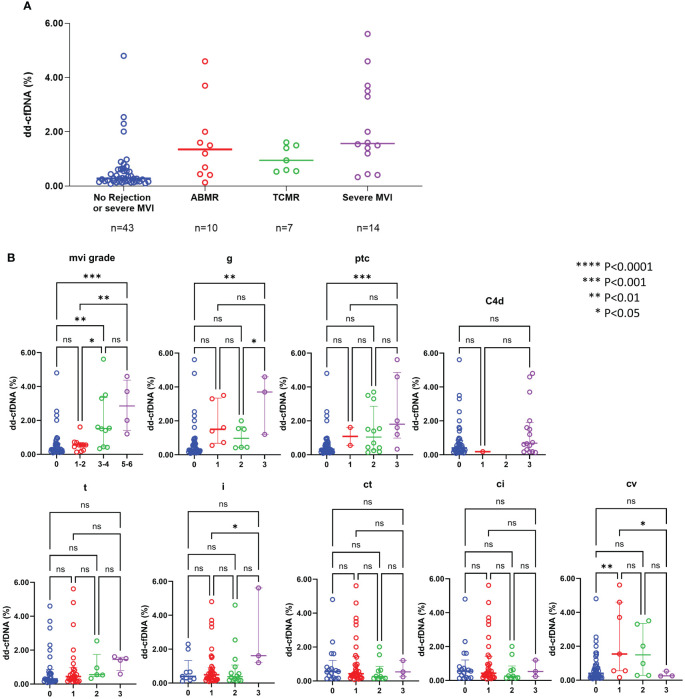
dd-cfDNA levels according to the rejection type **(A)** and individual histological lesions **(B)** ABMR, antibody-mediated rejection; TCMR, T-cell mediated rejection; MVI, microvascular inflammation; g, glomerulitis; ptc, peritubular capillaritis; t, tubulitis; i, interstitial inflammation; ct, tubular atrophy; ci, interstitial fibrosis; cv, arteriosclerosis; ns, not significant; *P<0.05, **P<0.01, ***P<0.001, ****P<0.0001.

The median level of dd-cfDNA was 1.35% (IQR: 0.43 – 2.43) in patients with ABMR, 0.95% (IQR: 0.55- 1.50) in patients with TCMR, and 1.57% (IQR: 1.01 – 3.55) in patients with severe MVI, and 0.32% (IQR: 0.17-0.70) in those without rejection, respectively. Increased dd-cfDNA levels were correlated with ABMR and severe MVI (**Figure 4A**). Aside from the obvious association with ABMR, the dd-cfDNA levels were correlated with the severity of g, ptc, and MVI score on allograft biopsy (P < 0.05) (**Figure 4B**). Especially, dd-cfDNA levels showed a strong correlation with MVI score (P < 0.001). Out of the 14 patients who developed severe MVI (defined as MVI = g + ptc ≥ 3) on allograft biopsy, 6 patients did not meet the criteria for ABMR, and they were also found to be negative for DSA. However, despite no ABMR and DSA, five patients of them showed elevated levels of dd-cfDNA, measuring 1.4% to 5.6%. Tubulointerstitial lesions on allograft biopsy did not show any correlation with dd-cfDNA levels (P < 0.05).

### 3.5 Predictive value of dd-cfDNA and DSA for biopsy-proven rejection

**Figure 5:**
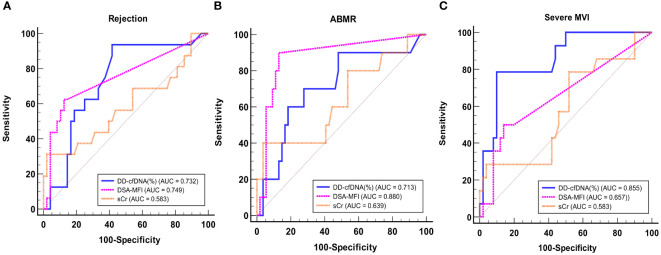
ROC curve analysis of dd-cfDNA, DSA-MFI, and sCr for detecting any rejection **(A)**, ABMR **(B)**, and severe MVI **(C)**. ABMR, Antibody-mediated rejection; MVI, microvascular inflammation; AUC, Area Under the Curve; DSA, donor-specific antibody; MFI, median fluorescence index; sCr, serum creatinine.

For the purpose of diagnosing BPR, ABMR and severe MVI, ROC curve analyses were performed (**Figure 5**). DSA-MFI exhibited superior Area Under the Curve (AUC) values compared to dd-cfDNA or serum creatinine (sCr) levels for identifying any type of rejection (AUC: 0.749 for DSA-MFI vs. 0.732 for dd-cfDNA and 0.583 for sCr). Conversely, for severe MVI detection, %dd-cfDNA levels presented a higher AUC value of 0.855, surpassing DSA-MFI (0.657) and sCr (0.583).

**Table 2** shows the diagnostic sensitivities and specificities for DSA and dd-cfDNA individually and in combination, utilizing cutoffs of 0.4% and 1.0% for dd-cfDNA. Combination of DSA and dd-cfDNA (>0.4%) showed sensitivities of 56.3% and 80.0% and specificities of 91.7% and 90.7% for detecting BPR and ABMR, respectively. For detecting severe MVI, dd-cfDNA (92.9%) showed a higher sensitivity relative to DSA (50.0%). In addition, dd-cfDNA (>1.0%) and combination of DSA and dd-cfDNA (>0.4%) exhibited improved specificity for severe MVI detection, reaching 90.0% and 88.0%, respectively. The positive predictive value (PPV) and negative predictive value (NPV) of dd-cfDNA (>0.4%) for severe MVI were 37.1% and 96.6%, respectively. At a dd-cfDNA threshold of >1.0%, PPV was 68.8% and NPV for severe MVI was 93.8%. For detection of severe MVI, DSA had a PPV and NPV of 38.9% and 84.8%, respectively, and DSA combined with dd-cfDNA (>0.4%) had a PPV and NPV of 53.9% and 86.3%, respectively.

**Table 2:** Diagnostic performance of DSA, dd-cfDNA, and combination of DSA and dd-cfDNA for detecting rejection, ABMR, and severe MVI.

	Rejection		ABMR		severe MVI	
	Sensitivity (%) (95%CI)	Specificity (%) (95%CI)	Sensitivity (%) (95%CI)	Specificity (%) (95%CI)	Sensitivity (%) (95%CI)	Specificity (%) (95%CI)
DSA	62.5 (35.4-84.8)	83.3 (69.8-92.5)	90.0 (55.5-99.8)	83.3 (70.7-92.1)	50.0 (23.0-77.0)	78.0 (64.0-88.5)
dd-cfDNA (>0.4%)	93.8 (69.8-99.8)	58.3 (43.2-72.4)	90.0 (55.5-99.8)	51.9 (37.8-65.7)	92.9 (66.1-99.8)	56.0 (41.3-70.0)
DD-cfDNA (>1.0%)	50.0 (24.7-75.3)	83.3 (69.8-92.5)	60.0 (26.2-87.8)	81.5 (68.6-90.7)	78.6 (49.2-95.3)	90.0 (78.2-96.7
DSA or dd-cfDNA (>0.4%)	100.0 (79.4-100.0)	50.0 (35.2-64.8)	100.0 (69.2-100.0)	44.4 (30.9-58.6)	92.9 (66.1-99.8)	46.0 (31.8-60.7)
DSA & dd-cfDNA (>0.4%)	56.3 (29.9-80.2)	91.7 (80.0-97.7)	80.0 (44.4-97.5)	90.7 (79.7-96.9)	50.0 (23.0-77.0)	88.0 (75.7-95.5)

DSA, donor-specific anti-HLA antibody; dd-cfDNA, donor-derived cell free DNA; ABMR, antibody-mediated rejection; MVI, microvascular inflammation.

## 4 Discussion

In this study, we found that the combination of DSA and dd-cfDNA showed synergistic performance for BPR diagnosis. In addition, our findings emphasize upon the highest predictive value of dd-cfDNA particularly for allograft microvascular injury, especially severe MVI. When predicting the risk of severe MVI, dd-cfDNA showed a higher predictive value than DSA alone or a combination of dd-cfDNA and DSA. This suggests that integration of the dd-cfDNA test into clinical practice could lead to improved early detection of severe MVI, ultimately resulting in better outcomes for KTRs.

Our initial step involved a comprehensive comparison of clinical and laboratory parameters between patients with high dd-cfDNA levels and low dd-cfDNA levels. By utilizing the value of dd-cfDNA (0.4%) as a threshold, patients were stratified into distinct high and low dd-cfDNA groups. There was no difference in demographics between the two groups, except DSA at the time of biopsy. In accordance with previous reports (9, 19), the high dd-cfDNA group had a higher proportion of DSA-positive patients. When analyzing the frequencies of rejection, the high dd-cfDNA group had 16 cases of rejection, consisting of 9 ABMR, 6 TCMR, and 1 mixed rejection case. The incidence of rejection was significantly higher in the high dd-cfDNA group (15/35, 42.9%) as opposed to the low dd-cfDNA group (1/29, 3.4%) (P<0.001). We also investigated the distribution of DSA and dd-cfDNA results within patients experiencing rejection. When focusing on ABMR cases, 10% demonstrated only DSA positivity with negative dd-cfDNA results, and an additional 10% demonstrated only dd-cfDNA positivity with negative DSA. Interestingly, 42.9% of severe MVI cases were MVR without DSA, and featured positive dd-cfDNA results (DSA/dd-cfDNA -/+) (**Figure 2**). These findings suggest a strong association between dd-cfDNA and severe MVI. Also, this finding supports previous reports indicating the potential clinical application of dd-cfDNA testing in the early detection of allograft injury (11, 13, 20).

In our study, 7 patients were diagnosed with TCMR. Among them, 2 was diagnosed with acute TCMR grade IA/IB, 2 with chronic active TCMR grade IA, and 3 with chronic TCMR grade IB. The dd-cfDNA values measured in each patient were as follows: the patient diagnosed with acute TCMR grade IA, 0.55%; the patients with chronic active TCMR grade IA, 0.53% and 0.95%; and the patients with chronic TCMR grade IB, 0.59%, 1.4% and 1.5%. However, patient with a dd-cfDNA value of 1.5% had a mixed rejection with acute ABMR, making it challenging to attribute the elevated dd-cfDNA solely to pure TCMR. The average dd-cfDNA values in the DART study were 0.2% for TCMR grade IA patients, and 1.2% for TCMR grade IB and IIA patients (12). In another study using the AlloSeq dd-cfDNA kit, a median dd-cfDNA value of 0.52% was reported for a total of 14 TCMR patients, which is consistent with the findings in our study (21). While our study demonstrated lower predictive value for TCMR compared to the results from the Trifecta study (13), this may be attributed to the limited small number of cases and the predominance of chronic TCMR and grade IA TCMR.

In our study, the incidence of ABMR was 15.6%, which was relatively high compared to the typical range of 3-12% for ABMR occurrence (22). Our study enrolled specifically targeted patients who underwent allograft biopsy when rejection was clinically suspected, such as when serum creatinine increased, proteinuria occurred, *de novo* DSA emerged, or preformed DSA rebounded. As a result, the sample size was relatively small, but it was expected to have a higher proportion of patients with rejection, especially those diagnosed with ABMR. Consistently, studies conducted on patients who underwent clinically indicated biopsies reported high incidences of ABMR, ranging from 15% to 38% (12, 13, 21, 23). Our study also showed that 28.1% (18/64) of patients were DSA-positive, which was higher than the proportion of DSA-positive patients in general KT recipients. Similarly, studies conducted on patients who underwent clinically indicated biopsies also found high frequency of DSA-positive patients, ranging from 42% to 60% (21, 23).

Plasma dd-cfDNA is released into the recipient’s bloodstream in the form of cfDNA fragments as a result of donor cell injury in the allograft (11). Therefore, it is expected that dd-cfDNA would reflect endothelial cell injury more sensitively compared to epithelial cells, as endothelial cells could directly release into the bloodstream. Therefore, we conducted a comparative analysis across different Banff score categories and found significant differences in the g score and ptc score according to the dd-cfDNA levels. Consequently, the MVI score, which is the sum of the g score and ptc score, also exhibited significantly higher results in patients with high dd-cfDNA levels.

MVI is a histological feature suggestive of ABMR. Active ABMR was defined by Banff 2018 as a MVI score of 2 or higher accompanied by serologic evidence (DSA positivity) (16). Recent studies have reported a significant number of patients with severe MVI in the absence of DSA, suggesting the need to distinguish it as a distinct phenotype (17, 24–27). Some researchers have proposed categorizing this condition as MVR to differentiate it from ABMR (17, 26, 27). Callemeyn J. et al. (26) claimed that missing self-induced non-humoral natural killer cell stimulation is the underlying mechanism of MVR. Additionally, Delville M, et al. (17) have reported an association between MVR and IgG antibodies targeting non-HLA antigens. Dd-cfDNA may have the potential to detect injury prior to detectable DSAs and possibly even beyond current classification of ABMR, which may not be sensitive enough to detect the early changes in severe MVI or MVR.

Next, to evaluate the predictive value of DSA and dd-cfDNA for BPR and severe MVI, we conducted an ROC curve analysis. DSA-MFI exhibited superior AUC values compared to dd-cfDNA or sCr levels for identifying any type of rejection. However, our study included patients who underwent allograft biopsy because rejection was clinically suspected, leading to relatively high prevalence of rejection and positive DSA. The high prevalence of rejection and positive DSA may have influenced the positive predictive value, potentially resulting in an overestimation compared to its actual diagnostic value. In contrast, for severe MVI detection, %dd-cfDNA levels presented an AUC value of 0.855 signifying its heightened sensitivity to this distinct allograft injury. These results suggest that combining %dd-cfDNA with DSA can improve the diagnostic accuracy.

In the context of severe MVI identification, dd-cfDNA (at 92.9% sensitivity) surpassed DSA (at 50.0% sensitivity). Furthermore, dd-cfDNA (>1.0%) and combination of DSA and dd-cfDNA (>0.4%) led to noteworthy improvements in the specificity for severe MVI detection at 90.0% and 88.0%, respectively. These results highlight the potential of multiplex approaches in improving our diagnostic capabilities for allograft rejection.

The dd-cfDNA can reflect renal tissue damage caused not only by DSA-mediated injury but also by other factors, including non-HLA antibodies. Due to its ability to detect damage to the renal tissue from various causes, dd-cfDNA may have a higher predictive power for severe MVI compared to DSA alone or in combination with dd-cfDNA. In the present study, two patients demonstrating severe MVI without ABMR and DSA showed elevated levels of dd-cfDNA. In specific conditions, %dd-cfDNA can be elevated even in the absence of allograft injury. In our study, we only measured %dd-cfDNA without the absolute values. %dd-cfDNA measures the fraction of donor-derived DNA within the total DNA fragments. Consequently, when the overall cfDNA decreases, the %dd-cfDNA can appear higher. This effect is particularly influenced by rapidly dividing and abundant cells like leukocytes. Therefore, in patients with leukopenia, %dd-cfDNA can be elevated without rejection. However, these two patients with elevated dd-cfDNA values in the absence of DSA and ABMR did not have leukopenia. Hence, we thought that the elevation of dd-cfDNA is related to severe MVI. The cause of allograft injury in these patients might be associated with factors such as non-HLA antibodies or non-humoral NK cell stimulations (17, 26). Recently, there have been many reports on DSA-negative patients with severe MVI who do not meet the diagnostic criteria for ABMR (17, 27). The prognosis of these patients is expected to be poorer compared to those without rejection. Therefore, the importance of predicting severe MVI through the use of dd-cfDNA is expected to be increasingly emphasized in the future.

In terms of the threshold value of %dd-cfDNA, many previous studies have reported 1.0% (12, 13, 20, 28). Dauber EM, et al. (29) have even proposed higher thresholds, while a few authors have suggested thresholds around 0.8% (23, 30, 31), which were still higher than those in our study. For instance, in the ADMIRAL study by Bu L et al. (14) using the Allosure dd-cfDNA kit, they suggested an optimal dd-cfDNA threshold of 0.69% for detecting rejection. In this study, the authors confirmed that an elevation of dd-cfDNA above 0.5% threshold, in combination with a deviation from baseline, correlated with rejection, emphasizing the importance of serial monitoring. Bu L. et al., also noted a strong association between dd-cfDNA <0.5% and “allograft quiescence,” indicating allografts free from clinical and subclinical injury (14). Another study using the Allosure dd-cfDNA kit, an optimal dd-cfDNA threshold of 0.74% was suggested for ABMR, with a sensitivity of 100% and specificity of 71.8% (23). Mayer, K.A et al. (8) reported an optimal dd-cfDNA threshold of 0.78% for ABMR. In the most recent study by Mantios E et al. (21), performing serial dd-cfDNA surveillance with the AlloSeq dd-cfDNA kit, they reported a high odds ratio for rejection at a threshold of 0.5%.

In our study, only 25.0% of the total patients had dd-cfDNA levels above 1%, with a median dd-cfDNA value of 0.49%. Furthermore, at a 0.4% threshold of dd-cfDNA for rejection had a sensitivity of 93.8% and a specificity of 58.3%, comparable to findings in other studies. Therefore, we categorized the patients into low and high dd-cfDNA groups based on the median dd-cfDNA value. We believe that differences in race and variations in body weight among the study populations may have influenced the variation in the dd-cfDNA threshold. It is important to note that Asian populations have been significantly underrepresented in most studies examining the utility of dd-cfDNA. Additionally, the small sample size in our study and differences in dd-cfDNA measuring methodologies may have contributed to the observed differences.

Our study has several limitations. First, it was a single-center study with a relatively small sample size. However, considering that the study population consisted of patients who underwent kidney transplantation and indication-based biopsies, the number of participants was acceptable. Also, the number of subjects was comparable to those in the other previously published studies (20, 29, 30). Second, we only measured the baseline dd-cfDNA level at the time of allograft biopsy. Analyzing the treatment response based on the trend of dd-cfDNA changes in patients diagnosed with rejection through serial testing could further expand the clinical application of dd-cfDNA. Third, in our study, we measured dd-cfDNA as a fraction of total circulating cell-free DNA. %dd-cfDNA is influenced not only by allograft damage but also by the recipient’s total cell-free DNA (31, 32). In other words, if the release of recipient DNA increases due to factors like infection or exercise, %dd-cfDNA may decrease. As reported by Oellerich M, et al. (32), absolute quantification of dd-cfDNA can help avoid false negative results and improve the predictive value of dd-cfDNA for allograft injury. Moreover, we excluded patients who received kidney transplants more than twice or those who were multiple organ transplant recipients due to methodological limitations. Recently, Pettersson et al. (33) presented data on a new NGS-based dd-cfDNA assay that can quantify dd-cfDNA from multiple grafts both *in vitro* using generated mixed samples and in a cohort of patients who had received two kidney transplants. Consequently, further research including patients with multiple transplants is necessary to confirm our findings.

In conclusion, dd-cfDNA levels were found to be a promising predictive biomarker for BPR, comparable to DSA. Combination of DSA and dd-cfDNA can further enhance the predictive value for BPR. In addition, dd-cfDNA is highly effective in detecting endothelial injury and it demonstrates superior predictive power for severe MVI compared to DSA. Further research should be performed to validate these findings in a larger cohort and to expand its clinical validity to an immune monitoring marker and a long-term prognostic marker. In addition, serial monitoring of dd-cfDNA may also be of interest for evaluating the therapeutic effect of novel treatment for ABMR, such as the anti-CD38 monoclonal antibody, Felzartamab, which are currently evaluated in clinical trials (34, 35).

## Data availability statement

The original contributions presented in the study are included in the article/Supplementary Material. Further inquiries can be directed to the corresponding authors.

## Ethics statement

The studies involving humans were approved by the local Institutional Review Board of Seoul St. Mary’s Hospital. The studies were conducted in accordance with the local legislation and institutional requirements. The participants provided their written informed consent to participate in this study.

## Author contributions

HDK: Formal analysis, Methodology, Validation, Writing – original draft. HB: Formal analysis, Investigation, Methodology, Validation, Writing – original draft. HHK: Data curation, Formal analysis, Investigation, Writing – review & editing. HL: Data curation, Writing – review & editing. SE: Data curation, Writing – review & editing. CY: Supervision, Writing – review & editing. YC: Investigation, Writing – review & editing. BC: Conceptualization, Writing – review & editing. E-JO: Conceptualization, Writing – review & editing.

## Supplementary material

Supplementary Figure 1 Venn diagram showing the number of patients diagnosed with biopsy-proven rejections or severe microvascular inflammation.
