# Supplementary figures and images for: Donor-derived cell-free DNA predicted allograft rejection and severe microvascular inflammation in kidney transplant recipients
Source: Front Immunol. 2024 Jul 9;15:1433918. doi: 10.3389/fimmu.2024.1433918 (PMC11263016; doi:10.3389/fimmu.2024.1433918)

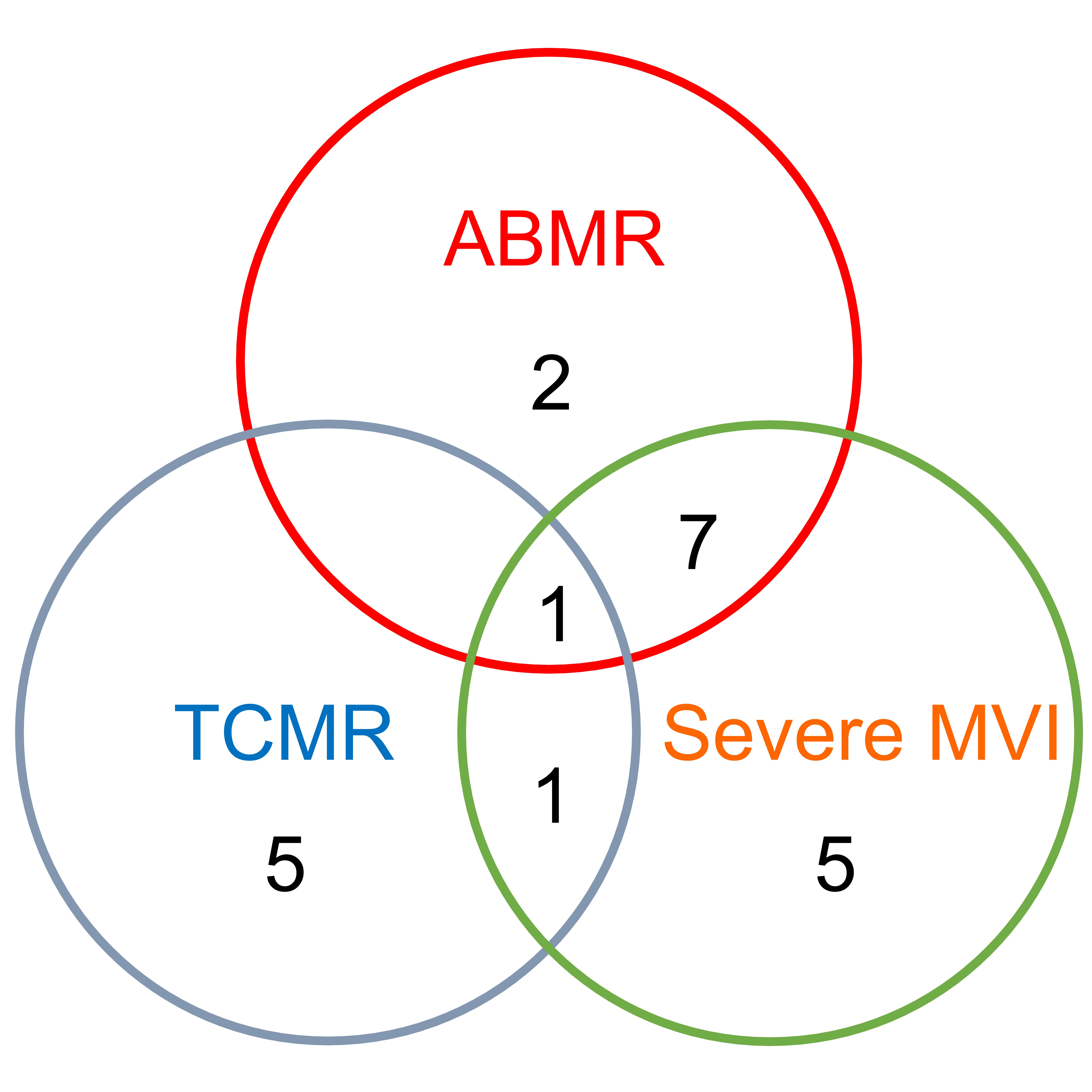

Supplement: Supplementary Figure 1 — Venn diagram showing the number of patients diagnosed with biopsy-proven rejections or severe microvascular inflammation. [file Image_1.jpeg]
